# Sensor Placement Strategies for Target Localization via 3-D TOA Measurements in Underwater Acoustic Sensor Networks

**DOI:** 10.3390/s26154793

**Published:** 2026-07-28

**Authors:** Rongyan Zhou, Weijie Tan, Meng Li, Baosheng Wang

**Affiliations:** 1 School of Information Engineering, Nanyang Institute of Technology, Nanyang 473004, China; zhoury619@163.com (M.L.); baosheng@126.com (B.W.); 2 Guizhou Provincial Laboratory of Big Data, Guizhou University, Guiyang 550025, China; wjtan@gzu.edu.cn

**Keywords:** time of arrival (TOA), underwater acoustic sensor networks (UASNs), Cramér-Rao lower bound (CRLB), optimal sensor placement, MinMax k-Means algorithm

## Abstract

This article investigates sensor placement strategies for 3-D time-of-arrival (TOA)-based target localization in underwater acoustic sensor networks (UASNs). To prevent the overestimation of localization performance common in idealized marine models, we derive an exact acoustic propagation time and estimate the TOA measurement variance using a non-linear ray acoustic model. Leveraging this formulation, we establish a realistic 3-D TOA measurement model that incorporates depth-dependent sound speed profiles (SSP) and spatially heterogeneous noise, where the trace of the Cramér-Rao lower bound (CRLB) serves as the optimization criterion. To solve the resulting non-convex optimization problem, we propose a MinMax *k*-Means algorithm to determine the optimal sensor configuration by minimizing the average of the trace of CRLB. Extensive numerical simulations demonstrate that the proposed placement strategy significantly enhances localization accuracy and robustness compared to conventional benchmarks, proving its effectiveness in complex underwater environments.

## 1. Introduction

Underwater Acoustic Sensor Networks (UASNs) have attracted considerable attention over the past few decades [1,2], driving the recent development of numerous potential applications, such as underwater security, ocean surveys, and submarine exploration [3,4]. Among various research topics in UASNs, target localization represents a fundamental capability, typically achieved by acquiring time, angle, and received signal strength measurements and executing corresponding localization algorithms [5,6,7].

Several localization techniques have been developed for UASNs, including time of arrival (TOA) and time difference of arrival (TDOA) methods, angle of arrival (AOA) techniques based on angle measurement, and received signal strength (RSS) based on signal energy measurement [8,9,10]. Among these, TOA-based localization offers several distinct advantages in underwater environments, making it the preferred choice for many underwater localization applications [11]. In recent years, extensive research has been conducted to improve the target localization accuracy in UASNs. Existing approaches can be broadly categorized into two groups: improving localization algorithms and optimizing sensor placement strategies. However, the majority of studies have focused on localization algorithms [12,13], while relatively few works have addressed sensor placement strategies. Given the complexity of underwater acoustic propagation, it is essential to incorporate the complex underwater acoustic channel characteristics when optimizing sensor placement [14,15]. Therefore, determining the optimal sensor placement in UASNs has become a critical research problem.

To address this challenge, the Cramér-Rao Lower Bound (CRLB) has been widely adopted as a performance metric for sensor placement optimization. The CRLB, formally defined as the inverse of the Fisher information matrix (FIM), quantifies the theoretical lower bound of localization estimation variance. Achieving the CRLB indicates that an estimator has attained the optimal localization performance. Consequently, CRLB-based criteria are widely adopted to formulate optimization frameworks for sensor placement [16,17,18]. In two-dimensional (2-D) space, the optimal sensor-target geometry for multistatic TOA localization was derived in [19], where the optimality criterion minimizes the area of the estimation confidence region, equivalent to maximizing the determinant of the FIM (D-optimality). Extending this approach to three-dimensional (3-D) scenarios, a novel optimization framework was formulated in [20] by minimizing the trace of the CRLB using TOA measurements. Furthermore, the concepts of maximum feasible angle and separation angle were introduced in [21] to transform the objective function and constraints into equivalent angular forms, thereby maximizing the determinant of the FIM to optimize range sensor placement in constrained regions. More recently, the authors in [22] proposed an efficient block majorization–minimization algorithm for optimal 3-D sensor placement, offering closed-form solutions and guaranteed convergence.

Although the aforementioned studies offer valuable sensor placement strategies, their optimization frameworks cannot be directly applied to the sensor placement problem in UASNs. In general, underwater acoustic measurement models are inherently complex, influenced by factors such as acoustic velocity profiles and multipath effects [23,24]. Furthermore, recent breakthroughs in physical boundary manipulations, such as the shallow-water waveguide with hybrid boundaries reported in [25], provide a crucial experimental foundation for practical underwater acoustic wave measurements. For instance, the authors in [26] proposed an optimal sensor placement method for 3-D underwater target localization using range-only measurements. However, their optimization process relied on idealized acoustic propagation models (e.g., constant sound speed), which significantly degrades localization robustness in realistic underwater environments. To address this limitation, a multivariate nonlinear optimization problem was formulated in [27] to minimize the trace of the CRLB incorporating a practical isogradient sound speed profile (SSP). Nevertheless, the resulting optimization problem was highly nonconvex, preventing guarantees of global optimality. Later, to enhance practicality, the effects of the SSP and the active sonar equation were integrated in [28] to improve coverage calculations and localization performance. However, the recommended five-node star configuration was costly and impractical for large-scale deployments. More recently, Huang [29] adopted the criterion of minimizing the trace of the CRLB to determine the optimal placement of reference nodes under Gaussian measurement noise, which correlates with the signal propagation path in 3-D underwater space. However, the computational complexity and adaptability of this method to large-scale or heterogeneous underwater networks remained unverified.

Most existing methods simplify the underwater acoustic propagation to a straight-line model. For instance, the studies in [20,27] optimized sensor placement based on standard Euclidean distances, assuming a constant sound speed. While mathematically tractable, such formulations ignore the vertical refraction of acoustic waves caused by depth-dependent sound speed variations. Although the approaches in [28,29] examine advanced spatial configurations or placement constraints, they fail to derive a closed-form FIM that directly incorporates 3-D bent-ray travel-time dynamics. This omission leads to significant performance degradation when the target moves across layers with highly variable sound speed gradients.

Distinct from these methodologies, our framework models the acoustic trajectory as a circular arc governed by the isogradient SSP. By establishing a direct mapping between the actual travel time and the 3-D optimal sensor placement, we derive an exact ray-tracing-based CRLB model. Furthermore, our proposed MinMax *k*-Means optimization algorithm directly targets the worst-case geometric dilution of precision (GDOP) by minimizing the maximum local trace of CRLB. This achieves substantially higher localization accuracy than conventional average-RMSE reduction methods. Overall, this paper focuses on optimal sensor placement for 3-D TOA-based target localization in UASNs. The main contributions of this work are summarized as follows:

- A comprehensive 3-D TOA measurement model is established by seamlessly incorporating the exact acoustic ray travel time and depth-dependent measurement variances derived from realistic underwater acoustic propagation mechanics.
- The analytical CRLB for 3-D TOA-based target localization under a realistic isogradient SSP is rigorously derived, with the trace of the CRLB serving as the optimization criterion to provide a theoretical foundation for performance evaluation.
- A customized CRLB-based MinMax *k*-Means optimization algorithm is proposed to solve the sensor placement problem by minimizing the maximum localized CRLB trace, ensuring guaranteed convergence and lower computational complexity than conventional non-convex optimization methods.
- Extensive simulation studies under both uniform and Gaussian target distributions validate the theoretical analysis, demonstrating that the proposed placement strategy consistently yields superior localization accuracy and enhanced robustness against environmental uncertainties.

The remainder of this paper is organized as follows: Section 2 delineates the 3-D TOA-based localization measurement model and formulates the minimax sensor placement optimization problem under physical constraints. Section 3 develops the core mathematical mechanics and the iterative framework of the proposed CRLB-based MinMax *k*-Means optimization algorithm. Section 4 provides extensive numerical simulations and Monte Carlo statistical trials to evaluate the performance of the proposed strategies against existing benchmarks. Finally, Section 5 concludes the paper and discusses future research directions.

## 2. System Model and Problem Formulation

In this section, we first introduce the TOA-based 3-D localization measurement model and the involved parameters. Subsequently, we adopt the isogradient SSP and varied noise measurement to quantify the localization error in terms of the CRLB.

### 2.1. System Scenario

In this paper, we consider a 3-D underwater acoustic localization scenario, where a stationary target is to be located using *N* cooperative sensors. Let C={1,2,3,…,N} denote the set of sensors, $q={\left[x,y,z\right]}^{⊺}\in R^{3}$ represent the position of the target, and $p_{i}={\left[x_{i},y_{i},z_{i}\right]}^{⊺}$ denote the location of the *i*-th sensor, where i∈C. The distance between the target and the *i*-th sensor is given by $r_{i}=∥q-p_{i}∥$, where ∥·∥ denotes the Euclidean norm.

Assuming that an acoustic signal generated at time $t_{0}$ arrives at the *i*-th sensor at time $t_{i}$, the signal arrival time in a noisy environment can be generally modeled as

(1) $$t_{i}=t_{0}+{\hat{t}}_{i}+\chi_{i}$$

where ${\hat{t}}_{i}$ denotes the actual noise-free acoustic propagation travel time from the target to the *i*-th sensor, and $\chi_{i}$ represents the measurement noise. Distinct from conventional straight-line propagation models that simplify this travel time as ${\hat{t}}_{i}=r_{i}/c$ under an idealized constant sound speed *c*, our framework explicitly derives ${\hat{t}}_{i}$ based on non-linear bent-ray tracing dynamics to prevent performance overestimation.

The measurement noise $\chi_{i}$ is assumed to be mutually independent and follows a zero-mean Gaussian distribution with variance $\sigma_{i}^{2}$. For notation brevity, we assume the signal emission time $t_{0}=0$. Consequently, the vector form of the TOA measurement model can be expressed as

(2) $$t=\hat{t}+\chi$$

with $t={\left[t_{1},t_{2},\ldots ,t_{N}\right]}^{⊤}$, $\hat{t}={\left[{\hat{t}}_{1},{\hat{t}}_{2},\ldots ,{\hat{t}}_{N}\right]}^{⊤}$, and $\chi ={\left[\chi_{1},\chi_{2},\ldots ,\chi_{N}\right]}^{⊤}$. The measurement noise covariance matrix is a diagonal matrix given by

(3) $$Q=\mathrm{cov}\left(\chi \chi^{T}\right)=\mathrm{diag}\left(\sigma_{1}^{2},\sigma_{2}^{2},\ldots ,\sigma_{N}^{2}\right)$$

Based on the observation vector t, the conditional probability density function (the likelihood function) of the TOA measurements given the target state vector u is rigorously formulated as

(4) $$f\left(t|u\right)=\frac{1}{{\left(2\pi \right)}^{N/2}\sqrt{\det(Q)}}e^{-\frac{1}{2}{\left(t-\hat{t}\right)}^{T}Q^{-1}\left(t-\hat{t}\right)}$$

The underwater sound speed is a hierarchical variable. Generally, it follows an isogradient depth-dependent SSP and can be expressed as

(5) c(z)=b+az

where c(z) is the exact local sound speed at the target depth *z*, *b* is the sound speed at the sea surface, and *a* denotes the steepness of the SSP, which is measured in 1/s (or $s^{-1}$). In realistic underwater environments, acoustic propagation is generally non-isotropic. The actual acoustic path does not propagate along a straight line but follows a curved trajectory [30]. According to Snell’s law, the deflection angle of this curved trajectory relative to the straight line connecting the target and the *i*-th sensor is given by

(6) $$\alpha_{i}=\arctan\frac{\sqrt{{\left(x-x_{i}\right)}^{2}+{\left(y-y_{i}\right)}^{2}}}{\frac{2b}{a}+\left(z+z_{i}\right)}$$

and the straight-line elevation angle of the line-of-sight (LOS) with respect to the horizontal plane is defined as

(7) $$\theta_{i}=\arctan\frac{z-z_{i}}{\sqrt{{\left(x-x_{i}\right)}^{2}+{\left(y-y_{i}\right)}^{2}}}$$

Consequently, the actual ray travel time (RTT) from the *i*-th sensor to the target along the bent curve is formulated as

(8) $${\hat{t}}_{i}=\frac{1}{a}\left[\ln\left(\frac{1+\sin(\theta_{i}+\alpha_{i})}{\cos(\theta_{i}+\alpha_{i})}\right)-\ln\left(\frac{1+\sin(\theta_{i}-\alpha_{i})}{\cos(\theta_{i}-\alpha_{i})}\right)\right]$$

### 2.2. Problem Formulation

The CRLB establishes a fundamental performance limit for any unbiased estimator in parameter estimation problems. Given an unbiased estimator $\hat{u}$ for the target state vector u, its covariance matrix is bounded from below by the CRLB matrix, which is mathematically defined as the inverse of the FIM

(9) $$E\left[\left(\hat{u}-u\right){\left(\hat{u}-u\right)}^{T}\right]\geq \mathrm{CRLB}\left(u\right)={\mathrm{FIM}}^{-1}$$

where the FIM characterizes the amount of information that the observable measurements t carry about the unknown parameter u. According to standard estimation theory, the FIM is evaluated by taking the mathematical expectation of the outer product of the log-likelihood score vector

(10) $$\mathrm{FIM}=E\left\{\left[\nabla_{u}\ln f\left(t|u\right)\right]{\left[\nabla_{u}\ln f\left(t|u\right)\right]}^{T}\right\}$$

By substituting the Gaussian log-likelihood function (4) into the statistical FIM definition (10) and evaluating the mathematical expectation over the zero-mean measurement noise, the stochastic formulation is deterministically simplified into the weighted summation of the spatial travel-time gradients:

(11) $$\mathrm{FIM}=\sum_{i=1}^{N}\frac{1}{\sigma_{i}^{2}}\left(\nabla_{u}{\hat{t}}_{i}\right){\left(\nabla_{u}{\hat{t}}_{i}\right)}^{T}$$

where $\nabla_{u}{\hat{t}}_{i}$ denotes the exact spatial gradient vector of the actual bent-ray travel time with respect to the target coordinates $u={\left[x,y,z\right]}^{T}$. To evaluate this gradient without relying on any conventional straight-line geometric approximations, we execute a rigorous analytical differentiation of the non-linear ray travel time Equation (8). First, let us introduce the horizontal projection distance $d_{i}$ and the horizontal azimuth angle $\psi_{i}$ between the target and the *i*-th sensor, defined respectively as

(12) $$d_{i}=\sqrt{{\left(x-x_{i}\right)}^{2}+{\left(y-y_{i}\right)}^{2}}$$

(13) $$\psi_{i}=arctan2\left(y-y_{i},x-x_{i}\right)$$

For notation brevity, we define an auxiliary depth-related parameter as

(14) $$V_{i}=\frac{2b}{a}+z+z_{i}$$

Thus, the deflection angle $\alpha_{i}$ in (6) can be compactly written as

(15) $$\alpha_{i}=\arctan\left(d_{i}/V_{i}\right)$$

By applying the multi-variable chain rule to the logarithmic RTT expression in (8), the spatial derivative of ${\hat{t}}_{i}$ with respect to the target coordinates $u={\left[x,y,z\right]}^{T}$ is formulated as

(16) $$\nabla_{u}{\hat{t}}_{i}=\frac{1}{a}\left[\frac{1}{\cos(\theta_{i}+\alpha_{i})}\left(\nabla_{u}\theta_{i}+\nabla_{u}\alpha_{i}\right)-\frac{1}{\cos(\theta_{i}-\alpha_{i})}\left(\nabla_{u}\theta_{i}-\nabla_{u}\alpha_{i}\right)\right]$$

Grouping the identical gradient vectors yields

(17) $$\nabla_{u}{\hat{t}}_{i}=\frac{1}{a}\left[\left(\frac{1}{\cos(\theta_{i}+\alpha_{i})}-\frac{1}{\cos(\theta_{i}-\alpha_{i})}\right)\nabla_{u}\theta_{i}+\left(\frac{1}{\cos(\theta_{i}+\alpha_{i})}+\frac{1}{\cos(\theta_{i}-\alpha_{i})}\right)\nabla_{u}\alpha_{i}\right]$$

Using trigonometric sum-to-product identities, the scalar coefficient terms are analytically simplified to

(18) $$\frac{1}{\cos(\theta_{i}+\alpha_{i})}-\frac{1}{\cos(\theta_{i}-\alpha_{i})}=\frac{2\sin\theta_{i}\sin\alpha_{i}}{\cos^{2}\alpha_{i}-\sin^{2}\theta_{i}}$$

(19) $$\frac{1}{\cos(\theta_{i}+\alpha_{i})}+\frac{1}{\cos(\theta_{i}-\alpha_{i})}=\frac{2\cos\theta_{i}\cos\alpha_{i}}{\cos^{2}\alpha_{i}-\sin^{2}\theta_{i}}$$

Substituting (18) and (19) back into the vector derivative formula gives the generalized gradient equation:

(20) $$\nabla_{u}{\hat{t}}_{i}=\frac{2}{a(\cos^{2}\alpha_{i}-\sin^{2}\theta_{i})}\left[\left(\sin\theta_{i}\sin\alpha_{i}\right)\nabla_{u}\theta_{i}+\left(\cos\theta_{i}\cos\alpha_{i}\right)\nabla_{u}\alpha_{i}\right]$$

Next, we evaluate the spatial gradients component by component for each individual axis. For the horizontal *x*-axis, by defining the horizontal distance $d_{i}=\sqrt{{\left(x-x_{i}\right)}^{2}+{\left(y-y_{i}\right)}^{2}}$, its partial derivative is $\frac{\partial d_{i}}{\partial x}=\cos\psi_{i}$. Applying the chain rule to (5) and (16) yields

(21) $$\begin{array}{cc} \frac{\partial \theta_{i}}{\partial x} & =-\frac{\sin\theta_{i}}{r_{i}}\cos\psi_{i}\\ \frac{\partial \alpha_{i}}{\partial x} & =\frac{\cos\alpha_{i}\sin\alpha_{i}}{r_{i}\cos\theta_{i}}\cos\psi_{i} \end{array}$$

Substituting the above spatial derivatives into the general gradient vector in (20) leads to an analytical cancellation of the complex term $\left(\cos^{2}\alpha_{i}-\sin^{2}\theta_{i}\right)$. Consequently, the *x*-component evaluates to

(22) $$\begin{array}{cc} \frac{\partial {\hat{t}}_{i}}{\partial x} & =\frac{2}{a(\cos^{2}\alpha_{i}-\sin^{2}\theta_{i})}\left[\left(\sin\theta_{i}\sin\alpha_{i}\right)\left(-\frac{\sin\theta_{i}}{r_{i}}\cos\psi_{i}\right)+\left(\cos\theta_{i}\cos\alpha_{i}\right)\left(\frac{\cos\alpha_{i}\sin\alpha_{i}}{r_{i}\cos\theta_{i}}\cos\psi_{i}\right)\right]\\ \frac{\partial {\hat{t}}_{i}}{\partial x} & =\frac{2}{a(\cos^{2}\alpha_{i}-\sin^{2}\theta_{i})}\left[-\frac{\sin^{2}\theta_{i}\sin\alpha_{i}}{r_{i}}\cos\psi_{i}+\frac{\cos^{2}\alpha_{i}\sin\alpha_{i}}{r_{i}}\cos\psi_{i}\right] \end{array}$$

By factoring out $\frac{\sin\alpha_{i}}{r_{i}}\cos\psi_{i}$, the complex term $\left(\cos^{2}\alpha_{i}-\sin^{2}\theta_{i}\right)$ analytically cancels out, and the *x*-component yields

(23) $$\frac{\partial {\hat{t}}_{i}}{\partial x}=\frac{2\sin\alpha_{i}}{ar_{i}}\cos\psi_{i}$$

Following the exact same symmetrical procedure for the *y*-axis, we obtain

(24) $$\frac{\partial {\hat{t}}_{i}}{\partial y}=\frac{2\sin\alpha_{i}}{ar_{i}}\sin\psi_{i}$$

For the vertical *z*-axis, the auxiliary depth parameter $V_{i}$ is defined in (14), and its vertical derivative is simply $\frac{\partial V_{i}}{\partial z}=1$. Applying the chain rule directly to the *z*-coordinate gives

(25) $$\begin{array}{cc} \frac{\partial \theta_{i}}{\partial z} & =\frac{\cos\theta_{i}}{r_{i}}\\ \frac{\partial \alpha_{i}}{\partial z} & =-\frac{\sin^{2}\alpha_{i}}{r_{i}\cos\theta_{i}} \end{array}$$

Substituting these terms into the *z*-component of (14) and factoring out $\frac{\sin\alpha_{i}}{r_{i}}$ yields

(26) $$\frac{\partial {\hat{t}}_{i}}{\partial z}=\frac{2}{a(\cos^{2}\alpha_{i}-\sin^{2}\theta_{i})}\frac{\sin\alpha_{i}}{r_{i}}\left(\sin\theta_{i}\cos\theta_{i}-\sin\alpha_{i}\cos\alpha_{i}\right)$$

To simplify this expression, we apply the trigonometric sum-to-product identities. The numerator translates to $\sin\theta_{i}\cos\theta_{i}-\sin\alpha_{i}\cos\alpha_{i}=\sin\left(\theta_{i}-\alpha_{i}\right)\cos\left(\theta_{i}+\alpha_{i}\right)$, and the denominator factors as $\cos^{2}\alpha_{i}-\sin^{2}\theta_{i}=\cos\left(\theta_{i}+\alpha_{i}\right)\cos\left(\theta_{i}-\alpha_{i}\right)$. By canceling the common term $\cos(\theta_{i}+\alpha_{i})$, the vertical gradient elegantly simplifies to

(27) $$\frac{\partial {\hat{t}}_{i}}{\partial z}=\frac{2\sin\alpha_{i}}{ar_{i}}\tan\left(\theta_{i}-\alpha_{i}\right)$$

To unveil the underlying physical mechanism, we establish a connection with the ray trajectory geometry. According to standard ray acoustics principles [31], in a medium with a constant sound speed gradient, the acoustic bent ray propagates precisely along a circular arc. Governed by Snell’s law, the radius of curvature of this arc is strictly defined as

(28) $$R_{i}=\frac{c(z)}{a\cos\gamma_{i}}$$

where c(z) is the localized sound speed at the target depth defined in (5), and $\gamma_{i}$ represents the local grazing angle at the target location. Geometrically, the straight-line distance $r_{i}$ between the target and the sensor acts as the chord of this circular arc, and the deflection angle $\alpha_{i}$ corresponds to the inscribed angle between the chord and the tangent. Thus, the exact geometric chord-length relation dictates that $r_{i}=2R_{i}\sin\alpha_{i}$. By rearranging this geometric identity and substituting (28), we obtain

(29) $$\frac{2\sin\alpha_{i}}{ar_{i}}=\frac{1}{aR_{i}}=\frac{\cos\gamma_{i}}{c(z)}$$

Geometrically, the tangent direction of the circular arc at the target depth exactly satisfies $\gamma_{i}=\theta_{i}-\alpha_{i}$. Substituting this physical identity back into the spatial derivatives, the exact travel-time gradient vector $\nabla_{u}{\hat{t}}_{i}$ is rigorously expressed without any straight-line approximations as

(30) $$\nabla_{u}{\hat{t}}_{i}=\frac{1}{c(z)}\left[\begin{array}{c} \cos\left(\theta_{i}-\alpha_{i}\right)\cos\psi_{i}\\ \cos\left(\theta_{i}-\alpha_{i}\right)\sin\psi_{i}\\ \sin(\theta_{i}-\alpha_{i}) \end{array}\right]$$

By substituting the derived gradient vector (30) into the statistical definition of the FIM in (11), the exact 3×3 FIM is derived mathematically by computing the outer product of the gradient vector with its transpose, weighted by the TOA measurement variance $1/\sigma_{i}^{2}$. Rather than relying on straight-line geometric approximations, the elements of this FIM explicitly incorporate the 3-D bent-ray dynamics—specifically, the local sound speed c(z) and the acoustic grazing angle $\left(\theta_{i}-\alpha_{i}\right)$.

Physically, the FIM quantifies the amount of spatial information the sensor network captures regarding the target’s position. The diagonal entries represent the absolute quantity of acoustic localization information projected exclusively along each respective Cartesian axis. The off-diagonal entries represent the cross-correlation of information between these axes. The symmetric FIM ($I_{jk}=I_{kj}$) is structured as

(31) $$\mathrm{FIM}=\left[\begin{array}{ccc} I_{xx} & I_{xy} & I_{xz}\\ I_{yx} & I_{yy} & I_{yz}\\ I_{zx} & I_{zy} & I_{zz} \end{array}\right]$$

where each distinct analytical element is constructed through the spatial projection of the effective ranging information

(32) $$\begin{array}{cccc} I_{xx} & =\sum_{i=1}^{N}\frac{\cos^{2}\left(\theta_{i}-\alpha_{i}\right)\cos^{2}\psi_{i}}{c^{2}\left(z\right)\sigma_{i}^{2}},\; & I_{yy} & =\sum_{i=1}^{N}\frac{\cos^{2}\left(\theta_{i}-\alpha_{i}\right)\sin^{2}\psi_{i}}{c^{2}\left(z\right)\sigma_{i}^{2}},\\ I_{zz} & =\sum_{i=1}^{N}\frac{\sin^{2}\left(\theta_{i}-\alpha_{i}\right)}{c^{2}\left(z\right)\sigma_{i}^{2}},\; & I_{xy} & =\sum_{i=1}^{N}\frac{\cos^{2}\left(\theta_{i}-\alpha_{i}\right)\cos\psi_{i}\sin\psi_{i}}{c^{2}\left(z\right)\sigma_{i}^{2}},\\ I_{xz} & =\sum_{i=1}^{N}\frac{\cos\left(\theta_{i}-\alpha_{i}\right)\sin\left(\theta_{i}-\alpha_{i}\right)\cos\psi_{i}}{c^{2}\left(z\right)\sigma_{i}^{2}},\; & I_{yz} & =\sum_{i=1}^{N}\frac{\cos\left(\theta_{i}-\alpha_{i}\right)\sin\left(\theta_{i}-\alpha_{i}\right)\sin\psi_{i}}{c^{2}\left(z\right)\sigma_{i}^{2}} \end{array}$$

Consequently, the resulting CRLB covariance matrix is obtained via $\mathrm{CRLB}\left(u\right)={\mathrm{FIM}}^{-1}$ using the algebraic cofactor inversion method. Specifically, this standard linear algebra technique calculates the exact analytical inverse by dividing the adjugate matrix by the determinant of the FIM. This step is crucial as it yields closed-form expressions for the spatial error variances on the principal diagonal. Based on this analytical inversion for the 3×3 matrix, the optimization criterion $T_{ar}$, defined as the trace of the CRLB matrix, can be evaluated in an exact closed form

(33) $$T_{ar}=\mathrm{Tr}\left({\mathrm{FIM}}^{-1}\right)=\frac{\left(I_{xx}I_{yy}-I_{xy}^{2}\right)+\left(I_{yy}I_{zz}-I_{yz}^{2}\right)+\left(I_{xx}I_{zz}-I_{xz}^{2}\right)}{\det(\mathrm{FIM})}$$

where the determinant det(FIM) is expanded as:

(34) $$\det\left(\mathrm{FIM}\right)=I_{xx}\left(I_{yy}I_{zz}-I_{yz}^{2}\right)-I_{xy}\left(I_{xy}I_{zz}-I_{xz}I_{yz}\right)+I_{xz}\left(I_{xy}I_{yz}-I_{yy}I_{xz}\right)$$

Given a narrowband acoustic signal characterized by a bandwidth of $B\left(\mathrm{Hz}\right)$, a center frequency of $f_{c}\left(\mathrm{Hz}\right)$, and a duration of $T_{s}\left(s\right)$, the theoretical CRLB for TOA estimation can be obtained as [32]

(35) $$\sigma_{i}^{2}\geq \mathrm{CRLB}\left(\mathrm{TOA}\right)=\frac{1}{8\pi^{2}BT_{s}f_{c}^{2}{\mathrm{SNR}}_{i}}$$

where ${\mathrm{SNR}}_{i}$ represents the received signal-to-noise ratio at the *i*-th sensor. Based on the analysis of underwater acoustic channel characteristics, the received signal-to-noise ratio at each sensor can be calculated once the system operating parameters are determined.

According to the classical passive sonar equation, neglecting the directivity of the acoustic transducer, the signal-to-noise ratio at the *i*-th sensor in UASNs is given by

(36) $${\mathrm{SNR}}_{i}^{s}=\mathrm{SL}-{\mathrm{TL}}_{i}-{\mathrm{NL}}_{i}$$

where SL represents the source level of the acoustic signal radiated by the target, ${\mathrm{TL}}_{i}$ denotes the propagation loss from the target to the *i*-th receiving sensor, and ${\mathrm{NL}}_{i}$ corresponds to the ocean ambient noise level at that sensor. Note that (36) is the decibel scale signal-to-noise ratio, which is converted to the linear power signal-to-noise ratio as

(37) $${\mathrm{SNR}}_{i}^{P}=10^{{\mathrm{SNR}}_{i}^{s}/10}$$

Substituting (36) and (37) into (35), the TOA estimation variance in the underwater environment is given by

(38) $$\sigma_{i}^{2}\geq \mathrm{CRLB}\left(\mathrm{TOA}\right)=\frac{1}{8\pi^{2}BT_{s}f_{c}^{2}{\mathrm{SNR}}_{i}^{P}}$$

The above Equation (38) reveals that the TOA estimation variance is fundamentally driven by marine propagation loss and ambient noise. Unlike the CRLB framework in [29], which relies on idealized straight-line propagation and uniform noise, our formulation accounts for realistic underwater acoustics. Specifically, we integrate the exact partial derivatives of the bent-ray travel time under an isogradient SSP using Snell’s law. Furthermore, we incorporate the passive sonar equation to dynamically model transmission loss and depth-dependent ambient noise. Consequently, the developed CRLB serves as a physically constrained metric that accurately characterizes the underwater channel.

## 3. Sensor Placement Solutions

In the preceding section, we established a comprehensive TOA measurement model for UASNs based on a realistic isogradient SSP and actual ray travel time. Subsequently, we systematically investigated the relationship between TOA estimation variance, propagation loss, and ambient noise. However, the aforementioned analyses are predicated on the impractical premise that the exact target location is known a priori. Since actual underwater target locations are inherently uncertain, optimizing a local CRLB for a single point is insufficient for practical placements. To ensure robust and consistent localization performance across the entire area of interest, we adopt the optimization criterion of minimizing the trace of the average CRLB over all possible target locations. The corresponding objective function is formulated as

(39) $$T_{AVG}=\frac{1}{\Omega }{\;\int \int \int \;}_{\Omega }T_{ar}\;dxdydz,$$

where Ω represents a 3-D spatial bounding volume corresponding to the test region. However, because the exact CRLB depends highly non-linearly on the target coordinates, deriving a closed-form analytical solution for this continuous triple integral over the entire volume Ω is mathematically intractable. The integral in (39) has no closed-form solution when substituting the explicit expression from (33).

### 3.1. The MinMax *k*-Means Optimization Algorithm

To facilitate a numerical solution, we discretize the continuous test region into a grid of candidate target locations. Under this discretization scheme, the optimization objective function for sensor placement is reformulated as

(40) $$\min_{p_{1},\ldots ,p_{N}}T_{AVG}=\min_{p_{1},\ldots ,p_{N}}\frac{1}{M}\sum_{m=1}^{M}\sqrt{T_{ar}\left(q_{m}\right)}$$

where *M* denotes the total number of discrete target points, $q_{m}$ is the *m*-th target position in the test region, and the vector $p={\left[p_{x,1},p_{y,1},p_{z,1},\ldots ,p_{x,N},p_{y,N},p_{z,N}\right]}^{T}$ concatenates the 3D coordinates of all *N* sensors.

Due to the highly non-convex nature of the objective function in (40), finding a global optimum is computationally intractable. To address this challenge, we introduce a customized MinMax *k*-Means algorithm that iteratively seeks a robust suboptimal placement. Specifically, we redefine the objective function to minimize the trace of the CRLB, derived from the non-linear bent-ray travel-time dynamics. Consequently, the algorithm update step replaces the conventional centroid-averaging strategy and iteratively solves a localized non-linear sub-optimization problem to dynamically search for sensor positions that minimize the theoretical localization error. The detailed implementation steps of the algorithm are outlined below:

1. Initialization
  The initial positions of *N* sensors are selected using the MinMax *k*-Means initialization strategy:
  - Randomly select a point in the test region Ω as the initial position of the first sensor $p_{1}$,
  - Select the point in the test region Ω farthest from $p_{1}$ as the initial position of the second sensor $p_{2}$,
  - For i=3…N: select the point that maximizes the minimum Euclidean distance to previously selected sensors.
2. Iterative Optimization
  The iterative framework is similar to the standard *k*-Means algorithm, but the update rule does not compute the centroid of each cluster. Instead, the position of each sensor is optimized individually to minimize the overall objective function. The detailed steps are as follows:
  - Allocation Step: Assign each virtual target point $q_{m}$ to its acoustically dominant sensor to form clusters. In 3-D target localization, a single TOA measurement cannot fully localize a target, and the corresponding 3×3 FIM (${\mathrm{FIM}}_{j}$) is rank-deficient. Consequently, an individual 3-D CRLB matrix is mathematically undefined. To resolve this, we leverage the fundamental duality between information and error. Instead of computing the CRLB, we utilize the trace of the individual FIM, denoted as $\mathrm{Tr}({\mathrm{FIM}}_{j})$, as a surrogate metric to strictly quantify the maximum spatial information contribution from a single sensor. Using the exact spatial gradients derived in (30), the individual FIM trace analytically evaluates to $\mathrm{Tr}\left({\mathrm{FIM}}_{j}\right)=\frac{1}{c^{2}\left(z\right)\sigma_{j}^{2}}$. Therefore, the target assignment is formulated to maximize this localized information contribution
    (41) $$C_{k}^{\left(t\right)}=\left\{q_{m}:k=\arg\max_{j}\mathrm{Tr}\left({\mathrm{FIM}}_{j}\left(q_{m},p_{k}^{\left(t\right)}\right)\right)\right\},\;k=1,\ldots ,K\;$$
    where $C_{k}^{\left(t\right)}$ denotes the set of target points assigned to the *k*-th sensor at iteration *t*. Here, the total number of clusters *K* is strictly defined to be equal to the total number of sensors *N* (i.e., K=N), ensuring that each optimized cluster centroid directly maps to the 3-D sensor location.
  - Update Step: For each cluster, k=1,…,K, if $\left|C_{k}^{\left(t\right)}\right|=0$, randomly generate a new position in the test region.Otherwise, fix the positions of all other sensors and update the position of the *k*-th sensor $p_{k}^{\left(t\right)}$ by solving the following sub-optimization problem numerically. Unlike the assignment step that evaluates individual information contributions using the rank-deficient single FIM, the update step optimizes the sensor position using the full-network configuration. This cooperative evaluation renders the global FIM full-rank and invertible, allowing us to transition from Tr(FIM) to the exact joint localization error bound Tr(CRLB)
    (42) $$p_{k}^{\left(t+1\right)}=\arg\min_{p\in \Omega }\;\sum_{q_{m}\in C_{k}^{\left(t\right)}}\;\mathrm{Tr}\left(\mathrm{CRLB}\left(q_{m};P_{-k}^{\left(t\right)}\cup \left\{p\right\}\right)\right)$$
    with $P_{-k}^{\left(t\right)}=\left\{p_{1}^{\left(t\right)},\ldots ,p_{k-1}^{\left(t\right)},p_{k+1}^{\left(t\right)},\ldots ,p_{K}^{\left(t\right)}\right\}$
  Mathematically, this update rule implements a block coordinate descent optimization scheme [33]. Specifically, we decompose the original high-dimensional optimization problem into *N* independent low-dimensional subproblems. For each subproblem, we fix the positions of all sensors except the *k*-th one, and adjust the position of the *k*-th sensor to minimize the sum of Tr(CRLB) over all virtual target points assigned to that sensor.
3. Convergence Judgment
  Terminate the iteration if the maximum position change of all sensors is less than a predefined threshold ϵ
  (43) $$\max_{k}\;∥p_{k}^{\left(t+1\right)}-p_{k}^{\left(t\right)}∥<\epsilon$$
4. Output
  Return the optimized sensor placement matrix $P^{\left(t+1\right)}$.

### 3.2. Mathematical Formulation and Mechanics of the Proposed MinMax *k*-Means Algorithm

To clearly isolate the architectural and physical innovations of the proposed optimization scheme, it is essential to contrast its fundamental mathematical mechanics against conventional geometric clustering algorithms.

1. Structural Comparison with Conventional Variants
  - Conventional *k*-Means: Minimizes the aggregate sum of squared Euclidean distances between data points and their respective cluster centroids. It operates under the implicit assumption of an isotropic, homogeneous spatial field, optimizing purely for geometric proximity
    (44) $$\min_{C,\mu }\sum_{k=1}^{K}\sum_{x\in C_{k}}{∥x-\mu_{k}∥}^{2}$$
  - Conventional MinMax *k*-Means: To prevent the formation of unbalanced clusters or empty cells, the conventional MinMax *k*-Means introduces a cluster-weighting mechanism to minimize the maximum intra-cluster variance in a feature space
    (45) $$\min_{C,\mu }\max_{k}\sum_{x\in C_{k}}{∥x-\mu_{k}∥}^{2}$$
    where x represents passive data samples, $\mu_{k}$ is the statistical cluster mean, and ∥·∥ is the coordinate-space Euclidean norm.
  - Proposed Underwater MinMax *k*-Means:
    Unlike conventional methods that cluster passive feature data based on Euclidean geometric distances, the proposed algorithm transforms the minimax framework for sensor placement. The core mathematical adaptations are defined as follows
    - Domain Adaptation: The metric space is transformed from the coordinate Euclidean domain into the Acoustic Information Domain regulated by the non-linear FIM.
    - Metric Adaptation: The traditional intra-cluster spatial variance is replaced by dual information-theoretic metrics derived directly from the 3-D bent-ray dynamics. Specifically, the assignment uses the individual information contribution ($\mathrm{Tr}({\mathrm{FIM}}_{j})$), while the update relies on the localized analytical CRLB trace ($T_{ar}$) evaluated over the joint network.
    - Objective Adaptation: The MinMax formulation is tailored to strictly minimize the worst-case localization error across all partitioned Voronoi subregions within the SSP waveguide
      (46) $$\min_{p_{k}}\max_{u\in V_{k}}\;\mathrm{Tr}\left(\mathrm{CRLB}(u;P_{-k}\cup \left\{p_{k}\right\})\right)$$
      where u represents the discrete target grid points within the *k*-th Voronoi cell $V_{k}$, and $u_{\mathrm{sensor},k}$ represents the optimized location of the *k*-th sensor. Note that while the cell $V_{k}$ is formed by maximizing the single-sensor information ($\mathrm{Tr}({\mathrm{FIM}}_{j})$), the minimax objective correctly minimizes the exact joint CRLB over the entire cooperative sensor network ($P_{-k}\cup \left\{p_{k}\right\}$).
2. Computational Complexity Analysis
  Let *M* denote the total number of discrete targets spanning the 3-D underwater test region, *N* represent the number of sensors to be placed, and *I* signify the total number of clustering iterations until convergence. The computational profile per iteration is evaluated through its two primary phases:
  - Assignment Phase: For each target grid point, the FIM contribution and its resulting analytical cofactor inversion ($T_{ar}$) must be evaluated across all *N* sensors. Utilizing the closed-form exact gradient derived in (11) and the 3×3 analytic matrix inversion in (31), the computational cost of evaluating the information contribution for a single grid-sensor pair is O(1). Thus, the total complexity for the assignment phase across the entire grid layout scales strictly as O(M·N).
  - Update Phase: The sensor locations are updated within their clustered subregions via a localized line-search or gradient-descent update to find the coordinate that minimizes the localized $T_{ar}$. For *N* independent sensors, this phase requires $O(N\cdot M_{k})$ operations, where $M_{k}\approx M/N$ is the average number of grid points per cluster, simplifies to O(M).
3. Convergence Characteristics
  Unlike conventional meta-heuristic algorithms that only provide probabilistic convergence guarantees, the proposed MinMax *k*-Means framework can be rigorously proven to converge to a stable local optimum based on the Monotone Convergence Theorem. Let the global MinMax localization error bound at the *t*-th iteration be defined as the optimization objective:
  (47) $$J^{\left(t\right)}=\max_{k}\max_{u\in V_{k}}T_{ar}^{\left(t\right)}\left(u,u_{sensor,k}\right)$$
  where $V_{k}$ denotes the *k*-th Voronoi subregion partitioned in the current iteration. The algorithm follows an alternating optimization paradigm with two sequential steps:
  - Assignment Step (fixed sensor locations): Each target grid point u is reassigned to the Voronoi cell corresponding to the sensor with the minimum trace of CRLB. This deterministic mapping guarantees non-increasing peak localization error in any subregion, satisfying
    (48) $$J_{\mathrm{assign}}^{\left(t\right)}\leq J^{\left(t\right)}$$
  - Update Step (fixed grid partitions): The sensor location is adjusted toward the local information-theoretic optimum within its corresponding subregion, which strictly reduces the regional maximum error bound. This step guarantees:
    (49) $$J^{\left(t+1\right)}\leq J_{\mathrm{assign}}^{\left(t\right)}$$
  Combining (48) and (49), the objective function sequence is monotonically non-increasing
  (50) $$J^{\left(t+1\right)}\leq J^{\left(t\right)}$$
  Given that the localization error metric $T_{ar}$ is bounded from below by the fundamental physical constraints of information retrieval limits ($J^{\left(t\right)}\geq J_{\min}>0$), the sequence must monotonically converge to a stable local optimum within a finite number of iterations.

## 4. Simulation Studies

In this section, comprehensive simulations are carried out to evaluate the performance of the proposed sensor placement strategies for UASNs. We first configure the simulation parameters, then employ the MinMax *k*-Means algorithm to optimize sensor placement, and finally a systematic comparison of RMSE performance is presented for three representative sensor placement strategies. All numerical simulations, ray-tracing computations, and optimization algorithms were implemented and executed using MATLAB R2023a.

### 4.1. Simulation Setup and Optimal Sensor Placement

The simulation parameters are configured as follows.

- Signal Parameters: The center frequency is set to $f_{c}=1000\;\mathrm{Hz}$, the sampling interval $T_{s}=0.001\;s$, the bandwidth B=600Hz, and the source level SL=180dB. The ocean ambient noise level is a random value in the interval of 60–70dB.
- Underwater Acoustic Propagation Parameters: The depth range of the acoustic target is set to 0–1000 m. The distance between the target and each sensor ranges from 0m to 1500m, and the acoustic emission angle ranges from −90° to 90°. The surface sound speed is b=1480m/s, and the underwater sound speed profile is assumed to be isogradient, increasing linearly with depth *z* as (5), and linear sound speed profile with steepness a=0.1.
- MinMax *k*-Means Algorithm Parameters: The target spatial probability follows either a uniform or a Gaussian distribution across the test region, depending on the specific evaluation scenario. The algorithm is terminated when the maximum number of iterations t=1000 is reached or the convergence condition ε=0.01 is satisfied.

**Figure 1:**
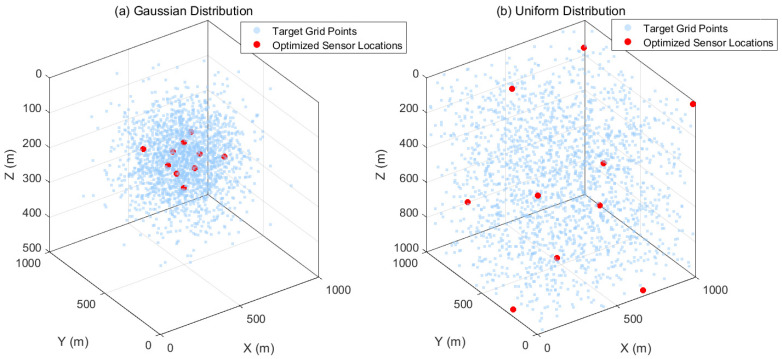
Optimized 3-D sensor placement under different target distributions with N=10: (**a**) Gaussian distribution; (**b**) Uniform distribution.

As illustrated in Figure 1, the proposed MinMax *k*-Means algorithm demonstrates the optimized placement of N=10 sensors under two different target distributions. Under the Gaussian target distribution, the algorithm clusters the majority of sensors around the high-probability hotspot ($\mu ={\left[500,500,200\right]}^{T}\;m$) to maximize localization performance. Simultaneously, a minority of sensors are placed at the periphery to constrain worst-case estimation errors at the boundaries. In contrast, under the uniform distribution scenario where targets are equally likely to appear anywhere in the test region, sensors are adaptively distributed across spatial extremities and deep-water environments. This placement strategy ensures robust coverage in zones characterized by severe acoustic propagation gradients.

Fundamentally, this stark contrast in spatial configurations originates from the underlying optimization mechanics. Rather than operating within the standard Euclidean coordinate space, the algorithm relies directly on the CRLB. Regardless of the underlying target distribution, the MinMax optimization criterion ensures that the worst-case trace of CRLB is strictly minimized. Driven by this strategy, the sensors can adaptively adjust their locations according to the target probability distribution, thereby achieving an optimal placement. This robust mechanism demonstrates that the proposed algorithm can reliably implement an optimized sensor placement in complex underwater acoustic environments, fully resilient to variations in target distributions or environmental uncertainties.

### 4.2. Localization Accuracy and Robustness Evaluation

In the following simulation, to comprehensively evaluate the effectiveness of the proposed placement strategy, we compare its localization accuracy against two state-of-the-art benchmark methods: the optimal 3-D TOA placement method based on a straight-line propagation model proposed by Xu et al. [20], and the CRLB-based reference sensor placement method designed for seafloor anchor positioning by Huang et al. [29]. To ensure a fair and rigorous comparison, a standard Maximum Likelihood Estimation (MLE) localization algorithm was implemented using the actual non-linear ray travel time model to obtain the estimated target coordinates. Consequently, the RMSE between the actual target positions and their estimated positions is calculated as follows:

(51) $$\mathrm{RMSE}=\sqrt{E\left\{{∥\hat{q}-q∥}^{2}\right\}}$$

All RMSE curves presented in the figures are evaluated over 1000 independent Monte Carlo trials for each target location configuration.

**Figure 2:**
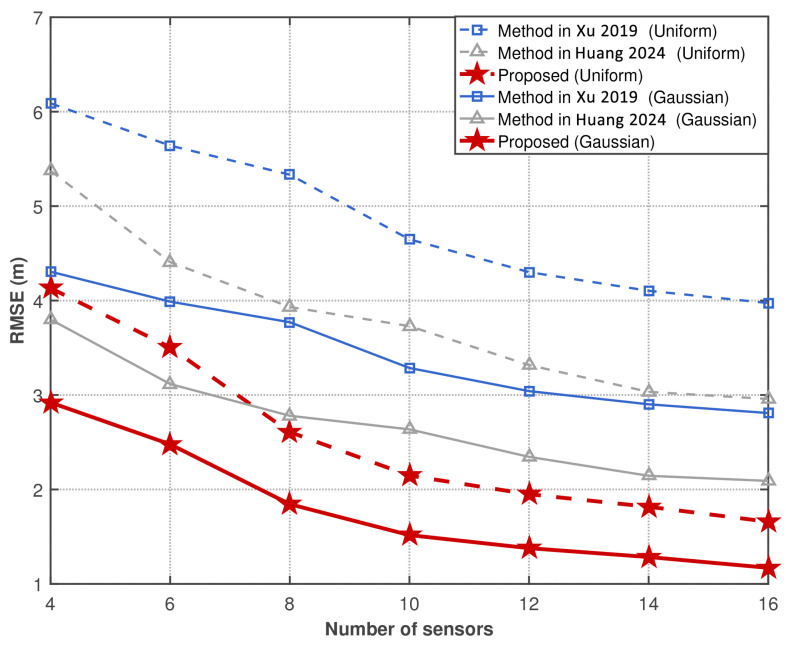
Comparison of localization RMSE versus the number of sensors for three different placement strategies with an SSP steepness of a=0.1 [20,29].

Figure 2 presents a comparative analysis of the localization RMSE versus the number of sensors for the proposed strategies and the state-of-the-art methods from [20,29]. As observed, although all RMSE curves exhibit a decreasing trend with an increasing number of sensors, a distinct performance gap exists between the two target spatial distributions. Specifically, the localization error under the Gaussian distribution is consistently lower than that under the Uniform distribution across all placement schemes. This fundamental difference arises because the Gaussian distribution provides a strong spatial prior, enabling the optimization algorithms to concentrate sensor resources around the high-probability region. In contrast, the Uniform distribution forces the network to broadly cover the entire test region, thereby elevating the average localization error.

Furthermore, the proposed MinMax *k*-Means algorithm consistently outperforms the existing benchmarks under both target distribution scenarios. Specifically, the method in [20] assumes a constant sound speed and straight-line signal propagation. This idealized assumption significantly deviates from realistic underwater acoustic ray bending, resulting in severe geometric mismatch. Similarly, although the scheme in [29] utilizes the CRLB, it fails to fully incorporate bent-ray traveling time dynamics. In contrast, by explicitly integrating depth-dependent acoustic characteristics and exact ray-tracing mechanisms into the optimization loop, the proposed method achieves the most robust and accurate network configuration, maintaining superior performance regardless of the underlying target distribution.

**Figure 3:**
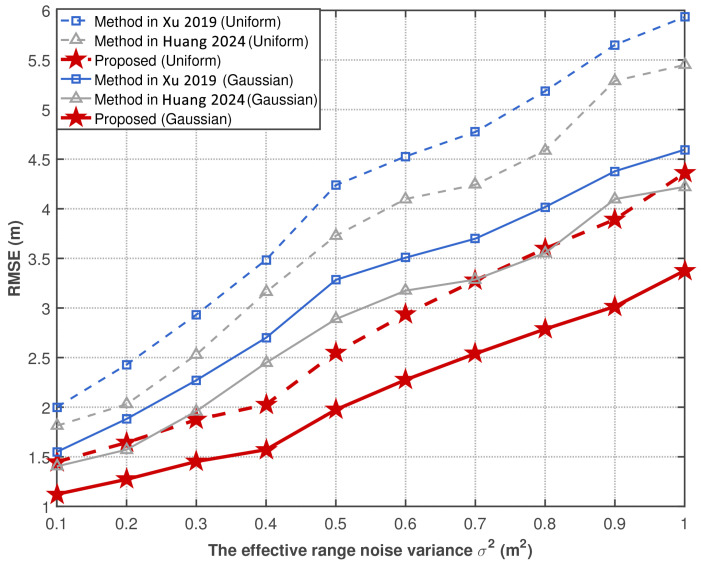
Impacts of the effective range noise variance on the localization performance under different placements with N=8 [20,29].

Figure 3 illustrates the impact of the effective range noise variance on the localization performance. The ambient ocean noise level $NL_{i}$ at each sensor is modeled as an independent uniform random variable within the interval U(60,70)dB. According to the passive sonar equation (36), the linear signal-to-noise ratio $SNR_{i}$ is calculated, directly determining the theoretical TOA measurement variance $\sigma_{i}^{2}$. To comprehensively evaluate system robustness, the effective range noise variance, defined via the local sound speed conversion as $c_{i}^{2}\sigma_{i}^{2}$, is systematically varied from $0.1\;m^{2}$ to $1.0\;m^{2}$ with the number of deployed sensors fixed at N=8.

The results explicitly reveal that the measurement noise exerts a more pronounced deteriorating effect on the Uniform distribution compared to the Gaussian distribution. In the Gaussian scenario, the centrally clustered sensor placement intrinsically provides higher redundant coverage and stronger geometric constraints for the majority of targets, effectively buffering the impact of severe noise variance. Conversely, the highly dispersed sensor placement necessitated by the Uniform distribution leaves peripheral targets highly vulnerable to noise fluctuations. Despite these severe environmental challenges, the proposed sensor placement strategy continuously achieves the lowest mean RMSE under both distributions. More importantly, as evidenced by the error bars derived from 1000 independent Monte Carlo trials, the proposed method exhibits the smallest standard deviation among all tested methods. This reduced statistical variability demonstrates that the physically-constrained, bent-ray CRLB optimization strictly prevents the formation of ill-conditioned network geometries, effectively mitigating extreme localization outliers even when TOA measurements are severely corrupted.

**Figure 4:**
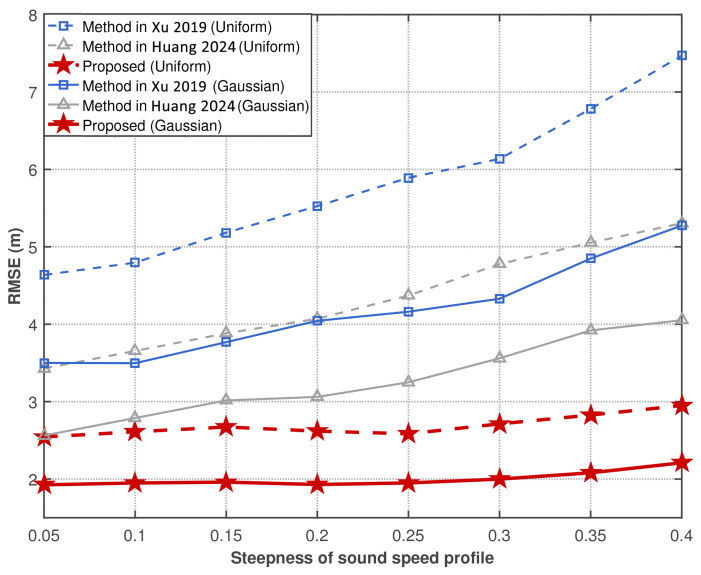
Impact of the SSP steepness on the localization RMSE for different placement strategies with N=8 [20,29].

Additionally, Figure 4 evaluates the impact of the SSP steepness (*a*) on localization performance. To clarify the experimental setup, the sensor placement was dynamically re-optimized for each specific SSP steepness *a*. A critical observation from this simulation is the divergent error growth rates among the methods. From a physical perspective, as the SSP steepness *a* increases, the acoustic ray bending becomes more severe, intrinsically increasing the difficulty and baseline error of target localization. Consequently, the RMSE of all methods exhibits an upward trend.

However, unlike the straight-line-based placement strategy in [20], which relies on a mismatched geometric assumption and therefore degrades rapidly, our proposed algorithm strictly integrates the exact non-linear bent-ray spatial gradients into its core optimization loop. Because the optimization objective explicitly depends on *a*, the proposed MinMax *k*-Means algorithm proactively shifts the sensor locations to optimally adapt to the changing acoustic environment. By dynamically adjusting the network geometry to avoid zones of high geometric dilution of precision (GDOP) induced by ray bending, the proposed strategy significantly mitigates the physical penalty of the steep SSP gradient. As a result, the RMSE growth is effectively suppressed, maintaining a much slower degradation and yielding superior robustness compared to the benchmark methods.

### 4.3. Monte Carlo Validation and Statistical Stability

**Figure 5:**
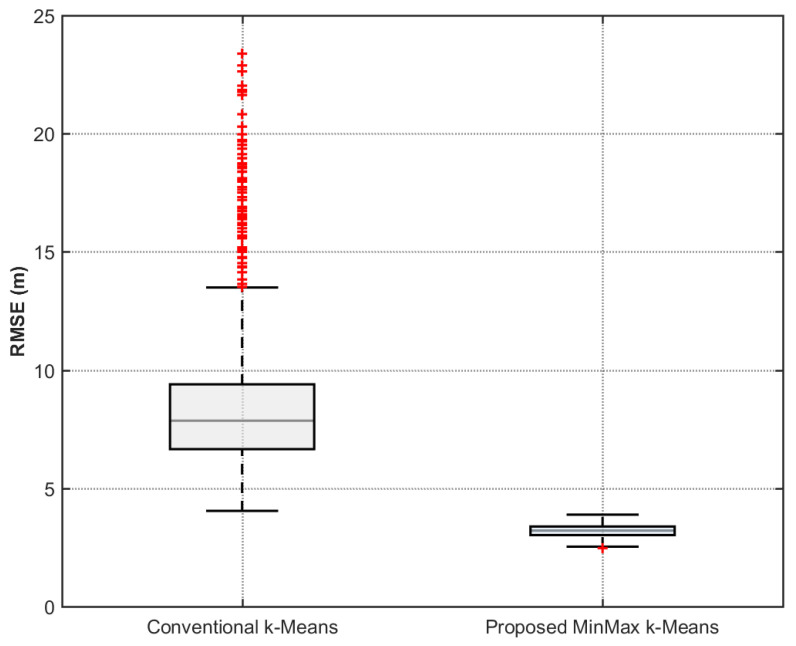
Boxplot of the maximum localization RMSE across 500 independent Monte Carlo trials.

Because partitioning algorithms are inherently sensitive to centroid initialization, and the 3-D TOA measurements in UASNs are continuously corrupted by stochastic noise, a rigorous Monte Carlo simulation was conducted to evaluate the statistical robustness of the proposed algorithm. To demonstrate the necessity of the CRLB-based objective, we compared our proposed method against a representative non-CRLB-based strategy, specifically the conventional *k*-Means algorithm, which optimizes sensor placement purely based on Euclidean geometric distances. We executed 500 independent trials, wherein both the initial sensor locations and the realizations of the zero-mean Gaussian measurement noise were fully randomized in each trial. As illustrated in Figure 5, the boxplot of the converged maximum localization RMSE reveals that the proposed MinMax *k*-Means strategy exhibits exceptionally low statistical variance.

Specifically, the interquartile range (IQR) of the maximum tracking error is tightly constrained to 0.36 m, and the median RMSE stabilizes at approximately 3.21 m. Notably, the proposed algorithm successfully bypasses severe local minima, effectively mitigating catastrophic localization failures. This compact statistical distribution provides compelling evidence that the derived optimization strategy yields a highly optimal spatial configuration, irrespective of the initial sensor placement.

Crucially, this robust statistical performance is strictly correlated with the theoretical convergence properties of the proposed algorithm. While a high dependency on initialization in conventional partitioning methods typically implies a susceptibility to local minima, the tightly bounded IQR observed in the MC trials confirms that the proposed optimization overcomes the stochastic measurement noise. Ultimately, these results demonstrate that the proposed MinMax *k*-Means algorithm robustly and monotonically steers the sensor placement toward a highly stable, near-global optimal configuration, explicitly preventing divergence and oscillatory behaviors.

## 5. Conclusions

This paper investigated optimal 3-D TOA-based sensor placement in UASNs by incorporating a realistic isogradient SSP and depth-dependent measurement noise. Utilizing the trace of the CRLB as the optimization criterion, a MinMax *k*-Means algorithm was developed to determine the optimal sensor placement. Extensive simulation results under both uniform and Gaussian target distributions demonstrate that the proposed strategy achieves superior localization accuracy and high robustness in dynamic underwater environments. Crucially, this strategy offers a theoretical pathway to streamline hardware deployment without sacrificing localization thresholds.

While this study establishes a foundational framework, it relies on a deterministic and perfectly known SSP. In realistic oceans, spatiotemporal fluctuations introduce SSP mismatches. Although our simulations on effective range noise variance partially demonstrate resilience to generalized ranging uncertainties, formally addressing SSP mismatch remains critical. Future work will incorporate stochastic SSP models to conduct comprehensive sensitivity analyses, and explore robust dynamic placement strategies for moving and multiple targets in uncertain environments.

## Data Availability

The data that support the findings of this study are available from the corresponding author upon reasonable request.
